# Apolipoprotein A (ApoA) in Neurological Disorders: Connections and Insights

**DOI:** 10.3390/ijms26167908

**Published:** 2025-08-16

**Authors:** Humam Emad Rajha, Ahmed Hassanein, Rowan Mesilhy, Zainab Nurulhaque, Nebras Elghoul, Patrick G. Burgon, Rafif Mahmood Al Saady, Shona Pedersen

**Affiliations:** 1 Department of Basic Medical Science, College of Medicine, QU Health, Qatar University, Doha P.O. Box 2713, Qatar; hr2003606@qu.edu.qa (H.E.R.); ah2309067@qu.edu.qa (A.H.); rm1903550@student.qu.edu.qa (R.M.); zm1902141@student.qu.edu.qa (Z.N.); ne2106561@student.qu.edu.qa (N.E.); 2 Department of Chemistry and Earth Sciences, College of Arts & Sciences, Qatar University, Doha P.O. Box 2713, Qatar; patrick.burgon@qu.edu.qa

**Keywords:** Apolipoprotein A, neurodegeneration, stroke, lipid metabolism, neuroinflammation, therapeutic targets

## Abstract

Apolipoprotein A (ApoA) proteins, ApoA-I, ApoA-II, ApoA-IV, and ApoA-V, play critical roles in lipid metabolism, neuroinflammation, and blood–brain barrier integrity, making them pivotal in neurological diseases such as Alzheimer’s disease (AD), stroke, Parkinson’s disease (PD), and multiple sclerosis (MS). This review synthesizes current evidence on their structural and functional contributions to neuroprotection, highlighting their dual roles as biomarkers and therapeutic targets. ApoA-I, the most extensively studied, exhibits anti-inflammatory, antioxidant, and amyloid-clearing properties, with reduced levels associated with AD progression and cognitive decline. ApoA-II modulates HDL metabolism and stroke risk, while ApoA-IV influences neuroinflammation and amyloid processing. ApoA-V, although less explored, is implicated in stroke susceptibility through its regulation of triglycerides. Genetic polymorphisms (e.g., *APOA1* rs670, *APOA5* rs662799) further complicate disease risk, showing population-specific associations with stroke and neurodegeneration. Therapeutic strategies targeting ApoA proteins, including reconstituted HDL, mimetic peptides, and gene-based approaches, show promise in preclinical models but face translational challenges in human trials. Clinical trials, such as those with CSL112, highlight the need for neuro-specific optimization. Further research should prioritize human-relevant models, advanced neuroimaging techniques, and functional assays to elucidate ApoA mechanisms inside the central nervous system. The integration of genetic, lipidomic, and clinical data offers potential for enhancing precision medicine in neurological illnesses by facilitating the generation of ApoA-targeted treatments and bridging current deficiencies in disease comprehension and therapy.

## 1. Introduction

Neurological diseases are among the foremost causes of morbidity and mortality worldwide, underpinned by complex pathophysiological mechanisms that involve lipid metabolism, inflammation, and neurovascular function [1,2]. Within this intricate framework, apolipoprotein A (ApoA) proteins, namely ApoA-I, ApoA-II, ApoA-IV, and ApoA-V, respectively, encoded by the *APOA1*, *APOA2*, *APOA4*, and *APOA5* genes, have emerged as pivotal players in disease development and progression. The ApoA proteins not only serve as potential biomarkers but also represent promising therapeutic targets for a range of disorders [3,4,5,6]. Among these, ApoA-I has garnered the most extensive attention in research due to its central role in cardiovascular protection. As the primary structural and functional component of high-density lipoprotein (HDL), ApoA-I facilitates reverse cholesterol transport (RCT), a key mechanism in removing excess cholesterol from peripheral tissues, and exerts anti-inflammatory effects, thereby contributing to overall homeostasis [7,8,9]. Clinically, ApoA-I is also used as a screening biomarker for hypercholesterolemia and cardiovascular risk assessment, with its levels aiding in the stratification of patients at risk for atherosclerotic cardiovascular disease and metabolic syndrome [10,11]. While ApoA-I dominates the protective functions of HDL, other members of the ApoA family, including ApoA-II, ApoA-IV, and ApoA-V, also play indispensable roles in lipid metabolism, vascular integrity, and immune regulation [6,12,13].

Despite the well-established protective effects of HDL and its associated proteins, recent advances in HDL-focused research have revealed the limited efficacy of therapies targeting HDL quantity in reducing neurological disease risk. Clinical trials have demonstrated that simply elevating HDL cholesterol (HDL-C) levels does not consistently translate into improved clinical outcomes [14,15]. Epidemiological studies [Single-Center Cohort, N = 15,784] and [Single-Center Cohort, N = 42,145] further complicate this narrative by uncovering a U-shaped relationship between HDL-C levels and all-cause mortality, suggesting that excessively high HDL-C may paradoxically increase health risks [16,17]. This shift in focus from HDL quantity to quality underscores the importance of understanding the HDL lipidome and proteome. Consequently, there is an urgent need to elucidate the specific contributions of individual ApoA proteins to disease mechanisms, as their distinct functions may hold the key to more effective therapeutic strategies [18].

ApoA-II, which constitutes 15–20% of the HDL proteome, plays a critical regulatory role in HDL metabolism. It influences cholesterol efflux, modulates immune responses, and interacts with enzymes involved in lipid metabolism [19,20,21]. Similarly, ApoA-IV, found in both chylomicrons and HDL, exhibits neuroprotective properties, along with anti-inflammatory and antioxidative effects [5,22]. Its ability to stabilize endothelial function and reduce oxidative stress highlights its potential as a therapeutic target. ApoA-V, present in small amounts in chylomicrons, very low-density lipoproteins (VLDLs), and HDL, is essential for triglyceride (TG) metabolism. Deficiencies in ApoA-V are strongly associated with hypertriglyceridemia, a significant risk factor for cardiovascular disease [23]. Despite growing recognition of their importance, the precise roles of ApoA proteins in disease pathogenesis remain incompletely understood. Genome-wide association studies (GWASs) and experimental models [Multi-Center Cohort, N = 1161], such as knockout and transgenic mice, have linked ApoA proteins to a spectrum of conditions, including stroke and neurodegenerative disorders like Alzheimer’s disease (AD) [23,24]. While these studies provide valuable insights into genetic variations affecting ApoA function, the biological and clinical implications of these findings continue to be actively explored.

This review provides a comprehensive analysis of the unique roles played by ApoA proteins in health and disease, emphasizing their potential as biomarkers and therapeutic targets. By dissecting their physiological and pathological contributions, we aim to illuminate their significance in the development and progression of neurological disorders. The multifaceted functions of ApoA proteins, from regulating lipid metabolism and maintaining vascular integrity to modulating inflammation and immune responses, underscore their importance as central mediators in disease mechanisms. Through this detailed exploration, we hope to offer fresh perspectives on the therapeutic potential of ApoA proteins, paving the way for innovative strategies to combat some of the most pressing health challenges of our time. Furthermore, understanding the interplay between ApoA proteins and other molecular pathways could unlock new opportunities for precision medicine, ultimately improving patient outcomes across diverse disease spectra.

## 2. Structure and Function of Apolipoprotein A (ApoA)

Apolipoprotein A proteins, including ApoA-I, ApoA-II, ApoA-IV, and ApoA-V, represent critical components of plasma lipoproteins that play a central role in lipid metabolism. These proteins are encoded by distinct genes, each imparting unique structural and functional characteristics that enable them to participate in a wide array of physiological and pathological processes. The amphipathic helical structures of ApoA proteins, which are essential for lipid binding and transport, arise from specific amino acid sequences arranged into α-helices [25]. This structural feature underpins their ability to interact with both hydrophobic lipids and hydrophilic aqueous environments, facilitating efficient lipid transport and metabolism. Such versatility highlights the emerging role of ApoA proteins in neuroprotection, blood–brain barrier (BBB) integrity, and the pathogenesis of neurodegenerative disorders.

Table 1 summarizes the major mechanistic roles of ApoA isoforms in neurovascular regulation, highlighting shared and unique pathways that have therapeutic relevance. Each protein exerts distinct and sometimes dual effects depending on microenvironment, concentration, and disease context, as shown via anti-inflammatory, pro-inflammatory, antithrombotic, antioxidant, and neuroprotective actions.

### 2.1. Introduction to ApoA Proteins

ApoA proteins are integral components of HDL and other lipoproteins, contributing significantly to lipid metabolism and brain homeostasis. Their structural diversity, particularly the presence of amphipathic helices, enables these proteins to bind and transport lipids effectively across the BBB. Among the ApoA family, ApoA-I is the most extensively studied due to its neuroprotective properties and its ability to modulate cholesterol efflux in the central nervous system (CNS) [41].

The amphipathic nature of ApoA proteins allows them to stabilize lipid-poor nascent HDL particles, which is particularly important for maintaining lipid balance in the brain parenchyma and cerebrospinal fluid (CSF) [42]. These amphipathic helices enable ApoA proteins to bind and transport lipids efficiently, a function that is essential for crossing the BBB and supporting lipid redistribution within the CNS. This process not only supports neuronal membrane integrity but also helps clear neurotoxic lipids implicated in neurodegenerative diseases. Furthermore, the structural flexibility of ApoA proteins enables them to interact with various CNS-specific enzymes and receptors, thereby modulating neuroinflammatory and oxidative stress pathways that are central to neurological disorders [43]. Additionally, ApoA proteins exhibit a dual role in inflammation, acting both as pro-inflammatory and anti-inflammatory agents depending on the physiological context. This dual functionality allows them to either promote immune responses to combat acute insults or suppress excessive inflammation to protect neural tissues, thus playing a critical role in maintaining CNS homeostasis [44].

### 2.2. Structure and Function of Apolipoprotein A-I

ApoA-I, the primary apolipoprotein in HDL, is encoded by the *APOA1* gene located on chromosome 11q23-q24 [3]. The preproprotein consists of 267 amino acids, predominantly synthesized in the liver (70%) and small intestine (30%), with emerging evidence of local production by astrocytes and microglia in the brain [45]. After proteolytic cleavage, the mature lipid-poor form (243 amino acids) circulates and crosses the BBB via specific transporters [46,47].

#### 2.2.1. Amphipathic Helical Structure

**Figure 1:**
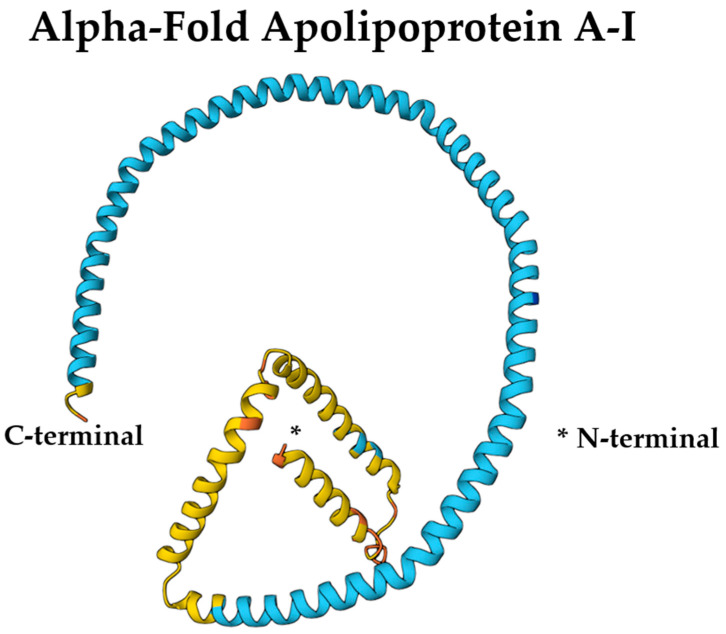
AlphaFold-predicted structure of ApoA-I (UniProt ID: P02647) colored by per-residue confidence (pLDDT). Blue = very high, cyan = high, yellow = low, orange = very low. Structure obtained from the AlphaFold Protein Structure Database (DeepMind and EMBL-EBI) under CC BY 4.0. * refers to the point of N-terminal end of the protein structure.

The amphipathic α-helices of ApoA-I are essential for its lipid-binding and solubilization capabilities within HDL particles [43]. These helices allow ApoA-I to interact with key enzymes such as lecithin-cholesterol acyltransferase (LCAT) and ATP-binding cassette transporter A1 (ABCA1), facilitating cholesterol efflux and esterification [42]. Specific segments of ApoA-I contribute uniquely to its functionality: residues 44–126 stabilize lipid-free ApoA-I, residues 144–185 activate LCAT, and residues 185–243 mediate ABCA1 binding [46]. Proline residues interspersed within a continuous 200-residue amphipathic helical segment promote curvature, enhancing ApoA-I’s structural adaptability and functional versatility [48]. Figure 1 illustrates the AlphaFold-predicted structure of ApoA-I (UniProt ID: P02647).

#### 2.2.2. Role in Reverse Cholesterol Transport (RCT)

**Figure 2:**
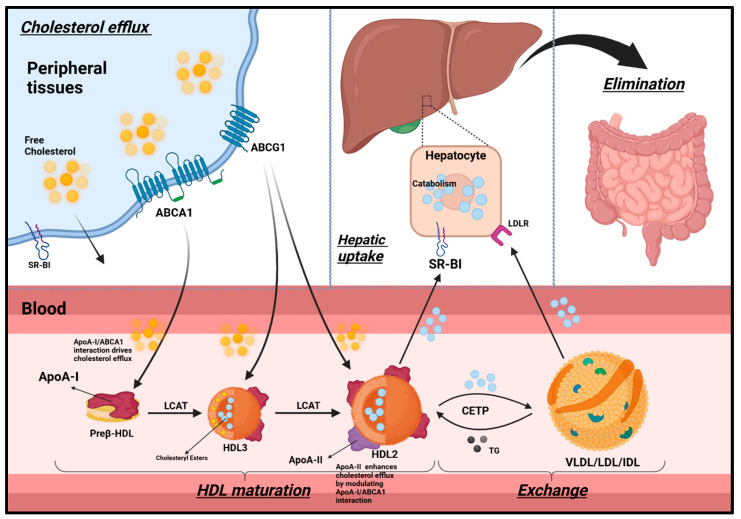
Retrograde cholesterol transport (RCT). HDL is classified into preβ-HDL, HDL3, and HDL2. Initially, ApoA-I is synthesized in the liver and intestines, which rapidly gathers cholesterol and phospholipids via ABCA1in circulation, forming discoidal/ preβ-HDL. Cholesterol efflux occurs passively via SR-BI and passive diffusion, or actively via ABCA1 and ABCG1. ABCA1 and ABCG1 are more significant as they account for 50% and 20% of cholesterol efflux, respectively. ApoA-I/ABCA1 interaction drives cholesterol efflux from peripheral cells. LCAT then esterifies the free cholesterol in the membrane to cholesteryl ester. Consequently, cholesteryl esters are relocated to the HDL core, helping with the maturation of a spherical HDL3. HDL3 can take up more cholesterol via ABCG1 and, with further LCAT activity, matures into larger HDL2 particles. Mature HDL2 particles can acquire additional ApoA-I and ApoA-II particles as they mature. ApoA-II enhances cholesterol efflux by modulating ApoA-I/ABCA1 interaction (further discussion in Section 2.2). ApoA-I on HDL2 interacts with SR-BI on hepatocytes to deliver cholesteryl esters. HDL2 can also exchange cholesteryl esters with VLDL, LDL, and IDL in return for triglycerides. These lipoproteins then deliver cholesterol to the liver via LDLR, where it is either catabolized and excreted in the bile or incorporated.

ApoA-I plays an indispensable role in RCT, a mechanism essential for clearing excess cholesterol from peripheral tissues and preserving cholesterol homeostasis within the brain, a process vital for neuronal function and neuroprotection [49]. During RCT, ApoA-I promotes the formation of nascent discoidal HDL particles, which subsequently mature into spherical HDL3 and HDL2 particles through a series of interactions with specific enzymes and receptors (Figure 2).

Synthesized in the liver and intestines, ApoA-I rapidly acquires cholesterol and phospholipids from peripheral tissues, including neurons and glial cells, through ABCA1-mediated efflux, resulting in the formation of nascent discoidal (preβ) HDL particles [45]. These particles are categorized into preβ HDL, HDL3, and HDL2 based on their apolipoprotein composition, molecular weight, and electrophoretic mobility [50].

Cholesterol efflux from cells occurs via both passive diffusion and receptor-mediated pathways. While SR-BI facilitates some of this transport, active mechanisms predominate: ABCA1 accounts for approximately 50% of total efflux, an especially critical pathway for amyloid-β clearance in AD, whereas ABCG1 contributes an additional 20% [51].

As RCT progresses, preβ HDL particles continue to interact with ABCA1, enabling further cholesterol acquisition [52]. The enzyme LCAT, with ApoA-I serving as a cofactor, esterifies free cholesterol within the phospholipid monolayer, facilitating the transformation of nascent HDL into mature, spherical HDL3 particles [53]. These HDL3 particles further enlarge into HDL2 as they accumulate additional cholesterol via ABCG1.

At the final stages of this maturation process, ApoA-I embedded in HDL2 interacts with SR-BI receptors expressed on brain endothelial cells and astrocytes, selectively delivering cholesteryl esters. These lipids support a range of essential neural functions, including neuronal membrane repair, synaptic plasticity, myelin maintenance, and neurosteroid synthesis [45].

Once delivered, cholesterol may be recycled for membrane biogenesis, metabolized into neurosteroids, or eliminated across the BBB. Excess cholesterol is stored in glial lipid droplets, while lipoprotein remnants engage low-density lipoprotein receptors (LDLRs) on neurons and glial cells to facilitate further redistribution as needed [45,54].

Beyond its role in cholesterol trafficking, ApoA-I-containing HDL particles undergo brain-specific remodeling by resident lipases. This adaptation optimizes their composition for efficient neuronal uptake and CSF circulation [55]. Collectively, this dynamic process underscores the central role of ApoA-I in preserving neural lipid equilibrium and sustaining effective neurotransmission and neuroprotection.

Finally, ApoA-I contributes additional protective functions (summarized in Table 1), including the following:

- Anti-ferroptotic activity: Enhances the NRF2/SLC7A11/glutathione (GSH) pathway in macrophages, increasing intracellular GSH levels and reducing lipid peroxidation, thereby protecting neural cells from ferroptosis [31].
- Antithrombotic support: Stabilizes PGI_2_ through its amphipathic α-helices, prolonging vasodilatory and antiplatelet effects, which may preserve BBB integrity and reduce micro-thrombosis during neuroinflammation [56].
- Synergistic anti-inflammatory effects: Interacts with HDL-associated molecules such as sphingosine-1-phosphate (S1P), paraoxonase, and clusterin to suppress CNS inflammation, particularly in the context of AD and MS [57].

These multifaceted roles reinforce ApoA-I’s significance as a key regulator of cholesterol trafficking, redox balance, and immune modulation in the CNS, highlighting its potential as a therapeutic target for neurodegenerative disorders, pending further in vivo and clinical validation.

#### 2.2.3. Anti-Inflammatory and Antioxidant Properties

Beyond its role in lipid transport, ApoA-I exhibits potent anti-inflammatory and antioxidant properties that significantly contribute to its neuroprotective effects [29,58,59,60,61]. These functions are essential for preserving brain homeostasis and may play a key role in slowing or preventing the progression of neurodegenerative disorders such as Alzheimer’s and PD [14]. Among its core mechanisms, ApoA-I inhibits TLR4-mediated neuroinflammation by preventing the receptor’s trafficking to lipid rafts, thereby suppressing downstream activation of NF-κB and the expression of tumor necrosis factor alpha (TNF-α) [26,27]. This pathway modulation is especially relevant in the context of chronic neuroinflammatory diseases.

In addition, ApoA-I confers protection against ferroptosis, a form of iron-dependent cell death, by activating the NRF2/SLC7A11/glutathione (GSH) axis. It upregulates NRF2 expression, which in turn enhances the production of SLC7A11 and intracellular glutathione, thereby mitigating lipid peroxidation in neurons and microglia [31].

ApoA-I also supports pro-survival signaling pathways. By modulating the PI3K/Akt and JAK2/STAT3 cascades, it promotes neuronal resilience and adaptive responses to cellular stress. Moreover, it stabilizes PGI_2_, a vasodilatory and antiplatelet molecule, contributing to cerebrovascular protection and reducing the risk of microthrombosis during neuroinflammatory states [44].

However, ApoA-I’s functional integrity can be compromised under inflammatory conditions in the CNS. MPO, released by activated microglia, catalyzes the nitration and oxidation of ApoA-I, leading to a reduced affinity for cholesterol efflux receptors such as ABCA1 and SR-BI [62,63]. In parallel, non-enzymatic post-translational modifications—including glycation, carbamylation, and oxidative alterations—further impair its antioxidant and antithrombotic activities [64].

These molecular modifications help explain why certain ApoA-I mimetic peptides and reconstituted HDL formulations, despite demonstrating efficacy in vitro, have failed to show consistent therapeutic benefits in clinical trials for neurological diseases. The evidence suggests that the functional quality of ApoA-I within the inflamed CNS microenvironment, rather than its absolute quantity, ultimately determines its neuroprotective and therapeutic potential [62,63].

In summary, ApoA-I plays a multifaceted role in neuroinflammation, cerebrovascular protection, and neuronal survival, primarily through its influence on pathways such as NF-κB, PI3K/Akt, and JAK2/STAT3 (Table 1). Its anti-inflammatory effects include inhibiting TLR4 trafficking in microglia and reducing TNF-α expression—key mechanisms in neurodegenerative diseases [26,28]. However, conflicting evidence regarding its context-dependent activity in neurological conditions (AD vs. PD) highlights the complexity of its actions. The antithrombotic and anti-ferroptotic effects demonstrate particular relevance for maintaining cerebral blood flow and protecting neurons from oxidative damage, although the precise mechanisms in the CNS require further elucidation [31]. The neuroprotective properties, including enhanced BBB integrity and reduced neuronal apoptosis, underscore its therapeutic potential for neurological disorders, but further validation through human studies is necessary due to the reliance on animal models and the unique biochemical environment of the human brain [32,65].

### 2.3. Structure and Function of Apolipoprotein A-II

ApoA-II is the second most abundant protein in HDL and influences lipid metabolism in both peripheral tissues and the CNS. It modulates HDL metabolism, cholesterol efflux, and neuroinflammatory responses [19,34].

#### 2.3.1. Homodimer Structure

ApoA-II is encoded by the *APOA2* gene located on chromosome 1q23.3. Initially synthesized as a 100-amino acid preproprotein, it undergoes proteolytic processing to yield the mature form, which has a molecular weight of 17.4 kDa [66]. The functional ApoA-II protein typically exists as a homodimer, composed of two identical 77-amino acid subunits, totaling 154 amino acids [67]. This configuration may facilitate its transport across the BBB.

**Figure 3:**
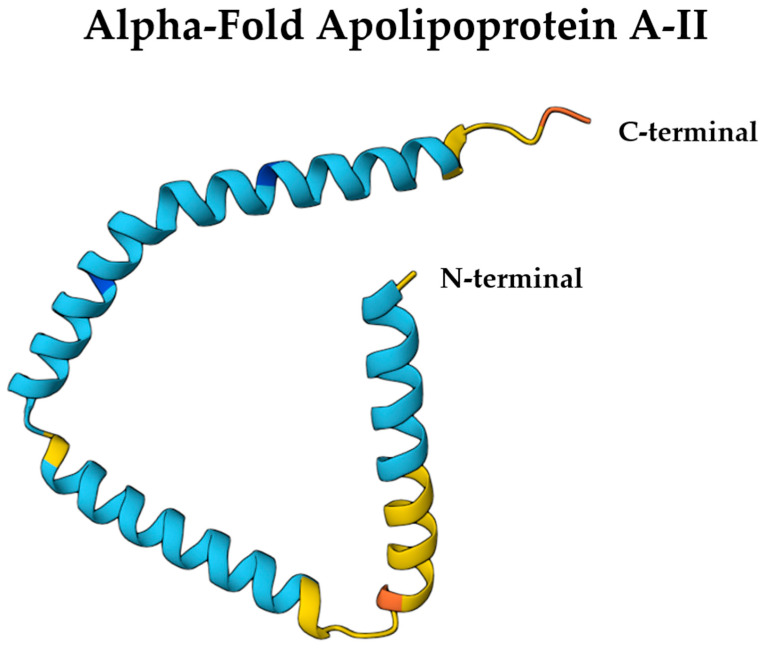
AlphaFold-predicted structure of ApoA-II (UniProt ID: P02652) colored by per-residue confidence (pLDDT). Blue = very high, cyan = high, yellow = low, orange = very low. Structure obtained from the AlphaFold Protein Structure Database (DeepMind and EMBL-EBI) under CC BY 4.0.

ApoA-II is a structurally compact apolipoprotein composed mainly of amphipathic α-helices, forming a disulfide-linked homodimer via a conserved cysteine at position 6 [68]. Each monomer adopts an α-helical conformation, and dimerization aligns the helices in an antiparallel fashion, facilitating interaction with HDL lipid surfaces. This quaternary structure imparts structural rigidity, limiting ApoA-II’s lipid exchangeability compared to ApoA-I [68]. AlphaFold-predicted structures (entry: P02652) reveal a helical hairpin topology consistent with lipid-binding roles, which can be visualized using the Mol* 3D viewer [69] (Figure 3). These features are critical for ApoA-II’s function in stabilizing HDL particles, modulating lipid homeostasis in the CNS, and potentially influencing BBB permeability in a conformation-dependent manner.

Synthesized primarily in the liver and intestines, ApoA-II circulates in plasma as a monomer, homodimer, or heterodimer, often in association with apolipoprotein D [70]. In the brain, ApoA-II interacts with ApoA-I on HDL particles, potentially regulating cholesterol homeostasis in neurons and glial cells [71]. These interactions are crucial for maintaining brain lipid balance and modulating neuroprotective HDL functions.

#### 2.3.2. Role in Lipid Metabolism

ApoA-II plays a complex role in lipid metabolism, particularly in regulating TG levels and modulating the relationship between ApoE and neurological disease risk. While lipid-poor ApoA-I primarily relies on ABCA1 for cholesterol efflux, lipidated HDL particles like HDL3 and HDL2 rely more on ABCG1 for maintaining brain cholesterol homeostasis, as shown in Figure 2.

The role of ApoA-II in cholesterol efflux is complex and has been the subject of conflicting evidence [21,72,73]. Some studies [Experimental Human Cells, N = 4] and [Experimental Animal Study, N = 35] suggest that ApoA-II enhances cholesterol efflux by inducing structural changes in ApoA-I, making it more effective at interacting with ABCA1 in neuronal and glial cells [74,75]. However, this effect is limited to a certain threshold of ApoA-II concentration, beyond which it may displace ApoA-I from HDL particles, potentially impairing lipid transport in the CNS.

ApoA-II can also increase ApoE and ApoC levels in HDL particles, which may inhibit lipoprotein lipase (LPL) and lead to higher TG levels [21]. In the brain, this interaction could influence lipid metabolism in astrocytes and microglia, potentially affecting neuroinflammatory responses. Additionally, ApoE can enhance VLDL production and secretion from the liver [21], a process that may indirectly impact lipid homeostasis in the CNS through the BBB. However, in ApoE-deficient conditions, ApoA-II might promote alternative lipid clearance pathways in brain cells by enhancing LDLR-mediated uptake of TG-rich lipoproteins [76]. This highlights the context-dependent nature of ApoA-II’s effects on brain lipid metabolism, which vary based on the presence of ApoE. The ApoA-II/ApoA-I interaction on HDL particles is crucial for cholesterol efflux in neurons and glia, particularly through ABCA1. However, excessive ApoA-II may displace ApoA-I, disrupting cholesterol transport in the brain and potentially contributing to neurodegenerative pathology [77].

Furthermore, ApoA-II has been implicated in modulating paraoxonase 1 (PON1) activity, although the evidence is conflicting. A study by Wang and colleagues [Experimental Animal Study, N = 38] showed decreased PON1 activity and increased oxidative stress in brain tissue in transgenic mice [73], while others in transgenic rabbits report increased PON1 activity with potential neuroprotective effects against lipid peroxidation [78]. The conflicting evidence regarding ApoA-II’s effect on PON1 activity in neurological contexts may be attributed to differences in animal models and brain-region-specific metabolism. To resolve these discrepancies, mechanistic studies employing neural cell cultures and biochemical assays can provide insights into how ApoA-II modulates PON1 activity in neurons and glia. Comparative studies across different models will help identify CNS-specific effects of ApoA-II on PON1 activity and its role in neuroprotection. Furthermore, clinical studies measuring CSF levels of ApoA-II and PON1 activity in neurodegenerative diseases could elucidate their relationship to cognitive decline and BBB integrity [66].

### 2.4. Structure and Function of Apolipoprotein A-IV

ApoA-IV is the largest apolipoprotein (46 kDa) and plays important roles in both peripheral lipid metabolism and CNS function. It is predominantly associated with postprandial chylomicrons and VLDL [22]. The APOA4 gene encodes a 396-residue precursor that undergoes post-translational cleavage to yield a functional ApoA-IV protein of 376 amino acids, primarily secreted from the small intestine and duodenum. ApoA-IV is also synthesized in brain regions, including the hypothalamus and brainstem, where it may influence feeding behavior and energy homeostasis [22].

#### 2.4.1. Structural Characteristics

**Figure 4:**
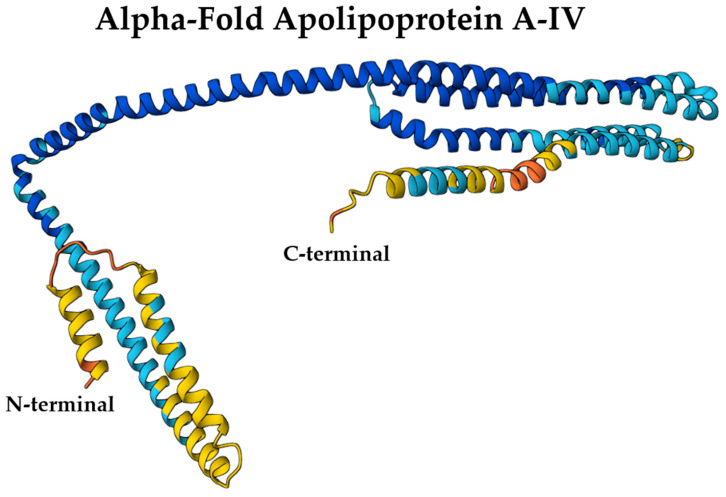
AlphaFold-predicted structure of ApoA-IV (UniProt ID: P06727) colored by per-residue confidence (pLDDT). Blue = very high, cyan = high, yellow = low, orange = very low. Structure obtained from the AlphaFold Protein Structure Database (DeepMind and EMBL-EBI) under CC BY 4.0.

Structurally, ApoA-IV shares similarities with the N-terminal structure of ApoA-I, characterized by the presence of amphipathic α-helical repeats that are crucial for lipid binding and self-association [22]. However, its helical bundles exhibit distinct structural differences. ApoA-IV consists of 22 amino acid peptide repeats that form amphipathic helices and self-assemble into homodimers in the absence of lipids [5]. The central core of the protein exhibits a well-organized structure, while the N- and C-termini display a comparatively less organized arrangement [5] (Figure 4).

#### 2.4.2. Biological Functions

ApoA-IV plays a multifaceted role with particular significance for brain health. While it facilitates TG metabolism in peripheral tissues through LPL activation, its CNS-specific isoforms contribute to lipid homeostasis in the brain [79]. ApoA-IV is a potent regulator of hypothalamic satiety signaling, influencing feeding behavior through direct actions on brainstem nuclei and dopaminergic reward pathways [22]. Its anti-inflammatory properties are especially relevant in the CNS, where it suppresses microglial activation and protects against neuroinflammatory damage [35] (Table 1). Furthermore, ApoA-IV is involved in β-amyloid clearance in the brain [80], suggesting a potential role in preventing or managing neurodegenerative diseases.

#### 2.4.3. Immunomodulatory and Vascular Roles

ApoA-IV exhibits complex immunomodulatory functions in the CNS, balancing both anti-inflammatory and pro-inflammatory effects. It suppresses microglial activation, reduces IL-17 and TNF-α production (key cytokines in neuroinflammation), prevents monocyte infiltration across the BBB, and decreases IL-6 levels [36]. Conversely, it enhances IL-10 and IFN-γ expression, suggesting context-dependent regulation of neuroimmune responses [36]. ApoA-IV demonstrates neuroprotective antioxidant capacity by preventing lipid peroxidation in brain-derived lipoproteins [36]. Through inhibition of NF-κB activation in brain endothelial cells and upregulation of DHCR24, it exerts anti-apoptotic effects on the neurovascular unit [36].

Furthermore, ApoA-IV acts as an S1P chaperone, maintaining S1P homeostasis and potentially impacting vascular integrity and neuroinflammation [81]. Its presence in endothelial cell-derived exosomes suggests a role in modulating endothelial function through post-translational modifications such as citrullination [82].

The involvement of ApoA-IV in DHCR24-dependent endothelial protection highlights its therapeutic potential, particularly in neurological diseases. This is due to DHCR24′s role in inhibiting NF-κB activation, which reduces vascular inflammation. Moreover, the ability of ApoA-IV to chaperone S1P could have significant implications for neurological diseases, given S1P’s major roles in vascular integrity, angiogenesis, immune cell trafficking, and neuroinflammation [83]. However, further mechanistic studies in human models are necessary to validate these findings and assess long-term benefits.

### 2.5. Structure and Function of Apolipoprotein A-V

ApoA-V is encoded by the APOA5 gene, yielding a protein with 366 amino acid residues, with the mature ApoA-V comprising 343 amino acids. ApoA-V biosynthesis occurs almost exclusively in the liver, with very minor contributions from the small intestine [23].

#### 2.5.1. Hydrophobic Structure

ApoA-V is characterized by a hydrophobic structure, primarily composed of amphipathic α-helix secondary structures. This hydrophobic nature renders it insoluble in aqueous solutions at neutral pH but facilitates its interaction with brain-specific lipoproteins [84]. The protein’s structure includes an N-terminal helix bundle (residues 1–146) that exhibits water solubility in the absence of lipids, a feature that may regulate its partitioning between CSF and brain parenchyma [84,85]. This unique structural feature is crucial for ApoA-V’s interaction with CNS lipids and lipoproteins, enabling it to modulate brain lipid metabolism and transport effectively [86].

**Figure 5:**
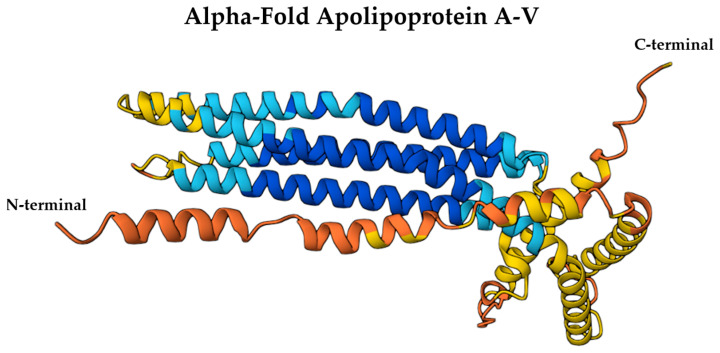
AlphaFold-predicted structure of ApoA-V (UniProt ID: Q6Q788) colored by per-residue confidence (pLDDT). Blue = very high, cyan = high, yellow = low, orange = very low. Structure obtained from the AlphaFold Protein Structure Database (DeepMind and EMBL-EBI) under CC BY 4.0.

Recent structural modeling, including AlphaFold predictions, reveals that ApoA-V also contains a C-terminal region rich in amphipathic α-helices, which may contribute to its lipid-binding specificity and affinity for TG-rich lipoproteins [87]. While the N-terminal helix bundle provides solubility in aqueous environments, the C-terminal domain appears more flexible and lipid-responsive, enabling insertion into lipoprotein surfaces or cell membranes under specific metabolic conditions [88]. This bipartite organization supports ApoA-V’s role as a lipid sensor and regulator within the neurovascular unit. Structural insights suggest that this configuration allows ApoA-V to act as a bridge between lipoprotein particles and lipases or receptors, such as glycosylphosphatidylinositol-anchored HDL-binding protein 1 and LPL, potentially influencing TG hydrolysis even within the brain [89]. Although no high-resolution crystal structures of full-length human ApoA-V are currently available, AlphaFold’s predicted model (UniProt ID: Q6Q788) offers a 3D structural framework that can be explored via the Mol* viewer to assess surface charge, hydrophobicity, and domain orientation, key features relevant to its CNS lipid trafficking functions (Figure 5) [69].

#### 2.5.2. Role in Lipid Regulation

ApoA-V plays a significant role in brain lipid regulation by interacting with lipoproteins, showing a preference for HDL in CSF [90]. Its solubility is enhanced in the presence of lipids, primarily due to the lipid-binding properties of its C-terminal end (residues 293–343) [90]. ApoA-V modulates lipid metabolism by interacting with the angiopoietin-like protein 3/8 complex (ANGPTL3/8), which normally inhibits LPL [91]. By suppressing the inhibitory activity of ANGPTL3/8, ApoA-V enhances LPL activity, leading to a decrease in plasma TG levels. This mechanism marks ApoA-V’s importance in maintaining lipid homeostasis [91].

#### 2.5.3. Anti-Inflammatory and Antioxidant Effects

In addition to its role in lipid metabolism, ApoA-V exhibits notable anti-inflammatory and antioxidant properties. Specifically, it inhibits TLR4-mediated NF-κB activation [38,92], which reduces TNF-α levels and regulates endoplasmic reticulum stress—mechanisms particularly relevant for neuroinflammatory conditions. Additionally, ApoA-V activates the PPARγ signaling pathway, decreasing reactive oxygen species (ROS) production and thereby mitigating oxidative stress, a key factor in neurodegenerative processes [93] (Table 1). While these effects have been primarily studied in hepatic and pulmonary contexts, their potential implications for neurological disorders warrant further investigation.

## 3. APOA Single Nucleotide Polymorphisms (SNPs)

SNPs in the genes encoding ApoA can significantly impact their structure and function, influencing lipid metabolism and neurological disease susceptibility [94]. These genetic variations may alter protein expression, stability, and interactions with lipids, potentially affecting neurovascular health and neurodegenerative processes.

The data in Table 2 summarize the complex role of ApoA gene polymorphisms in modulating neurological risk. Polymorphisms in APOA1, such as rs670, are associated with increased ischemic stroke risk. In contrast, certain APOA5 variants, notably rs651821, rs662799, and rs5069, show inconsistent associations with stroke and other neurological conditions. Some studies (e.g., case–control, N = 1656 [95]; meta-analysis, N = 79 [96]) suggest that the minor C-allele of rs651821 may offer protection, while others report that specific genotypes of rs662799 (AG/GG/CC) are associated with higher risk [95,97,98]. Meanwhile, rs5069 shows no significant association with cerebral amyloid angiopathy (CAA) [96,99]. Notably, rs2266788 in APOA5 modifies atorvastatin response in stroke patients [100] and exacerbates antipsychotic-induced hypertriglyceridemia in schizophrenia (SCZ) [101], underscoring its relevance in neuropsychiatric disorders.

Ethnic disparities further complicate these associations. For example, the rs662799 polymorphism demonstrates divergent stroke risk effects across populations [95,97,98], emphasizing the need for population-specific genetic studies. Additionally, linkage disequilibrium between variants (e.g., rs662799 and rs3135506) obscures their individual contributions, necessitating haplotype analysis and functional validation to clarify their roles in neurovascular diseases.

Mechanistic insights reveal how these SNPs may influence neurological outcomes. The rs2266788 polymorphism disrupts microRNA-3201 binding in the 3′ UTR of APOA5, increasing mRNA stability and expression, a potential pathway for dysregulated lipid metabolism in stroke or SCZ [101,102,103].

Together, these findings underscore the interplay of genetic, environmental, and lipid-mediated factors in neurological disorders, which rarely stem from a single variant. Neurologically focused studies are critical to disentangle these relationships, particularly for stroke, CAA, and neuropsychiatric comorbidities.

**Table 2:** ApoA gene polymorphisms and their associations.

Gene	Alleles	Implications in Neurological Diseases	Interpretation and Notes
APOA1			
rs670	C > A/C > T	Increased ischemic stroke risk (CT/TT) [95]. No association with CAA [99], PD [104], or stroke [105].	Minor A-allele may be protective for arterial stiffness but increases stroke risk.
rs5069	G > A	No association with CAA [99].	T-allele might be protective.
APOA5			
rs662799	G > A/G > C	Higher stroke risk (AG/GG/CC) [95,97,98]. Minor C-allele protects against stroke [96]. No association with AD [106,107]	Conflicting evidence, as some studies claim that the minor allele offers protection, while others associate it with greater risk.
rs2266788	G > A/G > C	Affects atorvastatin treatment effectiveness in stroke patients [100]. Schizophrenia: drug interaction with anti-psychotic treatment, higher TG levels with minor alleles [101]	C-allele disrupts microRNA-3201 binding, affecting APOA5 expression, and potentially increases risk of CAD and MI [103].
rs3135506	G > A/G > C	Higher stroke risk (CG genotype) [97], no association in some studies [108].	Minor C-allele might be associated with higher risk of cardiovascular diseases. Strong linkage disequilibrium with rs662799.
rs651821	C > A/C > T	Protective against ischemic stroke (C-allele) [96]	Mediation analysis shows a strong triglyceride link [109].

CAA, cerebral amyloid angiopathy. Note: Variations in the reported alleles of a polymorphism can occur depending on which strand of DNA is tested. We used the alleles reported by dbSNP. (>) This symbol indicates that the major allele (the one before the >) is substituted with the minor allele (the one after the >).

## 4. Apolipoprotein A-I

It is essential to distinguish between the lipoprotein and cholesterol pools in the plasma and those within the CNS [110]. The CNS contains a unique population of lipoproteins found in both the brain parenchyma and CSF, which differ markedly in composition and function from the well-characterized lipoproteins of the peripheral circulation, such as HDLs, VLDLs, LDLs, and chylomicrons [110]. While apolipoprotein E (ApoE) is considered the major apolipoprotein in brain lipoproteins, both ApoE and apolipoprotein A-I (ApoA-I) are significantly associated with CSF lipoproteins, underscoring their roles in lipid transport to the brain and neuronal tissues [110].

Astrocytes and microglia, key glial cell types, are capable of synthesizing ApoE and other apolipoproteins necessary for the assembly of brain-specific lipoproteins. However, ApoA-I is not synthesized by cells within the CNS and must be transported into the brain from the periphery via the blood–cerebrospinal fluid barrier at the choroid plexus. This extracerebral origin of ApoA-I has been confirmed through studies [Experimental Animal Study] involving mice engineered to lack ApoA-I production in the liver and intestines; these animals showed complete absence of ApoA-I in the CSF, demonstrating that the CNS does not generate this protein endogenously [111].

Once present in the CNS, ApoA-I becomes part of CSF lipoproteins and participates in the dynamic exchange between CSF and brain interstitial fluid. These lipoproteins are eventually cleared via the glymphatic system, which facilitates solute exchange between the CSF and brain tissue and returns metabolic waste to the systemic circulation. Given the specialized lipid and cholesterol demands of the CNS, these lipoproteins are uniquely adapted to support neural function and homeostasis. Notably, HDL particles are the only known peripheral lipoproteins capable of crossing the BBB, making them potential carriers for delivering ApoA-I and ApoA-II into the CNS [112,113]. As the primary protein components of HDL, these apolipoproteins may thus influence CNS lipid metabolism and neuroinflammatory processes.

In recent years, growing evidence has highlighted the involvement of ApoA proteins, particularly ApoA-I, in the pathogenesis and progression of several neurological disorders, including AD, PD, stroke, and MS. Their presence in the CNS, coupled with their known functions in lipid metabolism, inflammation modulation, and oxidative stress reduction, places ApoA proteins as key players in maintaining neural integrity and potentially influencing disease outcomes. A comprehensive summary of the roles of ApoA proteins (ApoA-I, ApoA-II, ApoA-IV, and ApoA-V) in these and other neurological disorders is provided in Table 3.

### 4.1. Apolipoprotein A-I in Alzheimer’s Disease (AD)

Alzheimer’s disease is primarily defined by the pathological accumulation of amyloid-β (Aβ42) plaques and neurofibrillary tangles, both of which drive chronic neuroinflammation and progressive neuronal dysfunction [138]. Emerging evidence has implicated ApoA-I in the pathophysiology of AD, with studies reporting reduced serum levels of ApoA-I in AD patients compared to healthy controls [118]. This decline suggests that dysregulation of ApoA-I may play a contributory role in the development or progression of AD.

Further investigations into the molecular profile of ApoA-I in AD have revealed not only quantitative reductions but also qualitative alterations. Protein analysis studies [Case-control study, N = 21] have identified conformational and structural modifications in ApoA-I among AD patients, potentially affecting its functional integrity [33]. These changes may compromise its capacity to support lipid metabolism and facilitate amyloid-β clearance, two processes critically involved in maintaining brain homeostasis and preventing neurodegeneration.

As the principal protein component of HDL, ApoA-I plays a central role in lipid metabolism through its involvement in RCT [33]. In this process, ApoA-I serves as a cofactor for LCAT, promoting the esterification of free cholesterol and enabling its transport from peripheral tissues to the liver for excretion [33]. This pathway is essential for cellular cholesterol homeostasis and has growing relevance in AD research, given the well-established interplay between lipid dysregulation and amyloid-β pathology.

The implications of ApoA-I levels differ depending on the biological compartment in which they are measured. In the systemic circulation, lower serum ApoA-I levels in AD patients suggest a potential protective function of this apolipoprotein against disease progression [122]. Reduced serum concentrations may reflect impaired lipid metabolism, diminished amyloid-β clearance, and heightened neuroinflammatory responses, collectively contributing to an environment conducive to AD pathogenesis.

In contrast, the relationship between CSF ApoA-I levels and AD appears more nuanced. Some studies [Experimental Animal Study, N = 63] indicate that elevated CSF ApoA-I levels correlate with slower cognitive decline in individuals with mild cognitive impairment, suggesting a possible compensatory or neuroprotective role within the CNS [122,123]. However, other findings show that higher CSF ApoA-I levels are associated with more advanced stages of AD, implying a potential maladaptive response or altered function under pathological conditions [139]. Within the CSF, ApoA-I likely contributes to localized lipid metabolism and amyloid-β handling, underscoring its multifaceted involvement in brain health and disease.

The interaction between ApoA-I and amyloid-β (Aβ) peptides is a key aspect of its potential involvement in AD pathophysiology. ApoA-I has been shown to bind directly to Aβ, promoting its clearance and reducing the formation of toxic plaques [124]. Experimental studies using mouse models [N = 100] have provided strong evidence for this protective role: increased serum levels of ApoA-I have been associated with reduced Aβ deposition and diminished neuroinflammatory responses [125]. Moreover, overexpression of ApoA-I in transgenic AD models has been linked to improvements in neurological function and memory performance, likely due to its ability to mitigate CAA [122].

In certain pathological conditions, such as when 27-hydroxycholesterol induces a decrease in ApoE levels, an increase in serum ApoA-I has been observed, suggesting a possible compensatory mechanism in response to ApoE deficiency [126]. Further supporting this hypothesis, intravenous administration of human recombinant ApoA-I Milano has been shown to significantly reduce Aβ accumulation in the brains of APP23-transgenic mice, a well-established model of AD [127]. These findings collectively suggest that ApoA-I-based interventions may offer a novel therapeutic strategy for AD by targeting both lipid metabolism and amyloid pathology, although clinical trials are necessary to confirm these effects in humans.

Reduced serum ApoA-I levels may also contribute to broader systemic metabolic disturbances that indirectly influence AD progression, potentially affecting both amyloid-β clearance and inflammatory processes in the brain [140]. In contrast, the local effects of ApoA-I within the CSF appear to be more directly involved in modulating Aβ deposition and neuroinflammation, which may in turn affect cognitive decline and overall disease severity.

While a number of studies consistently report lower serum ApoA-I levels in AD patients compared to controls, the relationship between CSF and serum ApoA-I remains less clear, with some findings appearing contradictory [140]. Notably, recent investigations have identified an inverse correlation between serum and CSF ApoA-I concentrations, suggesting that these two compartments may reflect distinct biological processes in AD [140]. This compartment-specific behavior implies that ApoA-I may exert differential roles depending on its location, highlighting the need for further research into its site-specific functions and regulatory mechanisms.

Longitudinal studies will be essential to determine whether changes in ApoA-I levels occur prior to the onset of AD or simply reflect disease progression. Additionally, exploring the interactions between ApoA-I and other lipid transport proteins, particularly ApoE, may reveal important compensatory pathways that could be leveraged therapeutically.

Given its central role in lipid metabolism and Aβ clearance, ApoA-I holds promise not only as a potential biomarker but also as a therapeutic target in AD. As a biomarker, fluctuations in ApoA-I levels, particularly in serum, could aid in early risk assessment or serve as a tool for monitoring disease progression. From a therapeutic standpoint, strategies aimed at enhancing ApoA-I expression or functional activity may help counteract AD pathology by improving cholesterol homeostasis, facilitating amyloid clearance, and dampening neuroinflammation. Encouraging results from animal models indicate that elevating ApoA-I levels can significantly reduce amyloid burden and inflammatory markers in the CNS [140], reinforcing the translational potential of ApoA-I-targeted therapies in the fight against AD.

### 4.2. Apolipoprotein A-I in Parkinson’s Disease (PD)

Parkinson’s disease (PD) is a progressive neurodegenerative disorder primarily characterized by the selective loss of dopaminergic neurons in the substantia nigra, widespread neurodegeneration, impaired autophagy, mitochondrial dysfunction, and the pathological accumulation of misfolded alpha-synuclein into intracellular aggregates known as Lewy bodies [141,142]. Emerging research has highlighted the potential involvement of ApoA-I in PD pathogenesis, particularly due to its ability to interact with alpha-synuclein, a protein central to PD pathology, and modulate its aggregation behavior.

ApoA-I demonstrates a protective role in PD through its capacity to alter the conformation of alpha-synuclein from a beta-sheet-rich structure, which favors fibril formation, to a more stable alpha-helical form [114,115]. This structural shift may prevent the formation of toxic oligomers and fibrils that contribute to neuronal damage. By stabilizing alpha-synuclein in a non-pathogenic state, ApoA-I could serve as an endogenous defense mechanism against the development and progression of PD [114,115]. Clinical evidence supports this hypothesis, as reduced levels of ApoA-I have been associated with earlier onset of PD and increased motor severity, suggesting its potential utility as a biomarker for disease progression [114,115]. 

Indeed, ApoA-I has garnered attention as a candidate biomarker in PD, with lower circulating levels correlating with greater vulnerability of the dopaminergic system. A meta-analysis across five independent PD cohorts revealed a significant association between reduced plasma ApoA-I concentrations and both earlier disease onset and more pronounced motor impairments [117]. These findings imply that monitoring ApoA-I levels may aid in early detection or stratification of PD risk and disease stage.

While ApoA-I appears to confer protection against PD-related motor pathology via its interaction with alpha-synuclein, elevated levels of the protein have also been linked to cognitive decline in some PD patients [143]. This paradoxical relationship underscores the complexity of ApoA-I’s role in PD and highlights the need for further investigation to clarify its functional contributions across different clinical phenotypes. Although its neuroprotective effects on dopaminergic neurons are promising, the impact of ApoA-I on cognitive outcomes in PD remains incompletely understood and warrants deeper exploration.

The interplay between lipid metabolism and PD pathophysiology is increasingly recognized, with literature suggesting that moderate hyperlipidemia may exert neuroprotective effects in PD by supporting neuronal membrane integrity and promoting neural repair [118]. As a key mediator of cholesterol and lipid transport, ApoA-I may play a central role in these processes, contributing to the preservation of neuronal function in the context of PD. Notably, dietary interventions rich in nicotinic compounds have shown limited effects on altering ApoA-I levels in PD patients, although stabilization of baseline ApoA-I concentrations was observed, suggesting a possible link between ApoA-I homeostasis and the mitigation of motor deficits [118]. Additionally, a negative correlation between ApoA-I levels and depression severity in PD patients has been reported, indicating a potential mood-protective effect of this apolipoprotein [118]. 

To fully understand the role of ApoA-I in PD, longitudinal studies are necessary to determine whether fluctuations in ApoA-I levels precede disease onset or reflect ongoing neurodegeneration. Investigating the interactions between ApoA-I and other lipid-associated proteins, such as ApoE, may reveal compensatory mechanisms that influence PD progression. Moreover, examining the effects of ApoA-I on mitochondrial function and proteostasis could open new therapeutic avenues aimed at preserving neuronal integrity in PD.

### 4.3. Apolipoprotein A-I in Multiple Sclerosis (MS)

Multiple sclerosis (MS) is a chronic autoimmune disorder of the CNS, characterized by inflammatory demyelination, axonal degeneration, and progressive neurodegeneration [144]. Recent studies have explored the potential role of ApoA-I in MS, focusing on its well-established anti-inflammatory and immunomodulatory properties. Given its central role in HDL-mediated cholesterol transport and its capacity to suppress inflammatory pathways, ApoA-I is increasingly being investigated for its neuroprotective potential in MS [131].

ApoA-I exerts its anti-inflammatory effects through multiple mechanisms, including the inhibition of pro-inflammatory cytokine production, suppression of oxidative stress, and promotion of immune tolerance. These properties suggest that ApoA-I may help mitigate the excessive immune activation and neuroinflammation that drive MS pathology [131]. Indeed, clinical observations indicate that individuals with progressive forms of MS exhibit significantly lower plasma ApoA-I levels compared to those with relapsing-remitting MS (RRMS) and healthy controls [128], pointing to a potential protective role of ApoA-I in disease stabilization and progression.

Genetic variation in the *APOA1* gene has also been implicated in MS outcomes. Specifically, the ApoA-I -75G/A promoter polymorphism has been associated with improved cognitive performance in MS patients, suggesting that this genetic variant may enhance ApoA-I expression and confer neuroprotection [130]. The precise mechanisms underlying this association remain under investigation, but they may involve enhanced lipid transport, anti-inflammatory signaling, or direct effects on myelin integrity.

Although the exact molecular pathways linking ApoA-I to MS are not yet fully elucidated, several lines of evidence support its potential as both a biomarker and a therapeutic target. Lower systemic levels of ApoA-I have been correlated with greater disease activity and higher disability scores in MS patients [145], reinforcing the notion that ApoA-I status could serve as a valuable indicator of disease severity and treatment response.

Further research is required to delineate the full spectrum of ApoA-I’s functions in MS, particularly its interactions with other components of lipid metabolism and its influence on CNS inflammation. Of particular interest is the potential role of ApoA-I in regulating oligodendrocyte function and promoting remyelination. Targeting ApoA-I pathways could offer novel strategies for enhancing myelin repair and reducing neurodegeneration in MS, opening promising avenues for future therapeutic development [146].

### 4.4. Apolipoprotein A-I in Stroke

Stroke remains a leading cause of morbidity and mortality worldwide, with ischemic and hemorrhagic subtypes accounting for the majority of cases. Emerging evidence has underscored the involvement of ApoA-I in stroke pathophysiology, particularly through its well-established roles in lipid metabolism and athero-protection [134]. Given its central function in HDL structure and activity, ApoA-I is increasingly recognized for its protective effects against cardiovascular and cerebrovascular diseases.

Elevated levels of ApoA-I have been consistently associated with a reduced risk of ischemic stroke, likely due to its capacity to promote RCT, reduce oxidative stress, and inhibit inflammatory pathways, key mechanisms that contribute to atherosclerotic plaque stabilization and vascular health [134]. However, the relationship between ApoA-I and hemorrhagic stroke appears more complex; some studies suggest that higher ApoA-I levels may paradoxically increase the risk of hemorrhagic stroke, underscoring the divergent effects of this apolipoprotein across stroke subtypes [133]. These findings highlight the need to consider stroke heterogeneity when evaluating the clinical implications of ApoA-I levels.

In addition to ApoA-I, ApoA-II has also been implicated in stroke risk. The FIELD trial found that elevated ApoA-II levels correlated with a lower incidence of all stroke subtypes, suggesting that it too may confer athero-protective benefits. Notably, ApoA-II has been shown to reduce circulating C-reactive protein (CRP) levels, an established marker of systemic inflammation, further supporting its role in mitigating inflammatory processes that contribute to cerebrovascular events in certain populations [133]. 

Beyond the classical ApoA proteins, genetic variants in related apolipoproteins have also been linked to stroke susceptibility. For instance, the rs662799 polymorphism in the *APOA5* gene has been associated with an increased risk of ischemic stroke [133]. This variant, located in the promoter region of *APOA5*, influences ApoA-V expression levels and is known to affect TG metabolism. Individuals carrying this allele often exhibit reduced ApoA-V levels and consequently elevated TG, a well-documented risk factor for atherosclerosis and ischemic stroke [133].

Collectively, these findings illustrate the multifaceted role of the ApoA family in stroke pathogenesis. While ApoA-I and ApoA-II appear to exert largely protective effects, the influence of genetic variants such as those in ApoA-V highlights the complexity of lipid metabolism in cerebrovascular disease. Understanding how these apolipoproteins interact with one another and with other lipid transporters, such as ApoE, may reveal compensatory mechanisms that modulate stroke outcomes and inform future therapeutic strategies.

### 4.5. Apolipoprotein A-I in Cerebral Amyloid Angiopathy (CAA)

CAA is characterized by the accumulation of amyloid-β (Aβ) peptides within the walls of cerebral blood vessels, leading to vessel fragility and increased risk of hemorrhagic complications [147]. Recent studies have explored the potential role of ApoA-I in CAA, focusing on its ability to assist in the removal of Aβ from the brain [147].

The potential function of ApoA-I in facilitating Aβ clearance from the brain may play a significant role in CAA. Studies have shown that a lack of ApoA-I exacerbates CAA and cognitive impairment, highlighting its protective effects against amyloid deposition [147]. In contrast, overexpression of ApoA-I in mice, driven by its native promoter in organs like the liver and intestine, has been linked to reduced CAA, diminished neuroinflammation, and improved cognitive function [147]. This suggests that ApoA-I could serve as a therapeutic target for managing CAA.

The absence of ApoA-I has been associated with increased cortical amyloid deposition, CAA, and inflammation in areas such as the cortex and hippocampus, particularly in the APP/PS1 mouse model [147]. This accentuates the importance of ApoA-I in maintaining brain lipid homeostasis and modulating neuroinflammation.

While the exact mechanisms by which ApoA-I affects CAA are not fully understood, its potential as a therapeutic target is promising. Further research is needed to clarify the role of ApoA-I in CAA and to explore its therapeutic applications in reducing amyloid deposition and improving cognitive outcomes.

Understanding the interplay between ApoA-I and other cholesterol transport proteins, such as ApoE, could provide insights into potential compensatory mechanisms in CAA [147]. Additionally, exploring the effects of ApoA-I on vascular integrity and amyloid clearance may offer new therapeutic avenues for CAA management.

### 4.6. Apolipoprotein A-I in Amyotrophic Lateral Sclerosis (ALS) and General Paresis

Amyotrophic lateral sclerosis (ALS) is a progressive neurodegenerative disorder characterized by the loss of motor neurons, leading to muscle weakness and paralysis [148]. Recent studies have explored the potential role of ApoA-I in ALS, focusing on its anti-inflammatory properties [149]. The incorporation of ApoA-I into endothelial cells has been shown to promote their survival by activating the PI3K/Akt signaling pathway, suggesting a potential protective mechanism against ALS [32]. Epidemiological studies indicate that higher circulating levels of HDL and ApoA-I are associated with a reduced risk of ALS onset [150]. Additionally, proteomic analysis of CSF from ALS patients following autologous bone marrow-derived mesenchymal stem cell therapy revealed increased ApoA-I levels, raising questions about whether ApoA-I elevation in CSF reflects a compensatory response, disease progression, or an effect of the treatment itself [151].

Beyond ALS, ApoA-I has also been implicated in general paresis. General paresis, a neuropsychiatric manifestation of late-stage syphilis, results in cognitive impairment. Serum levels of ApoA-I were found to be lower in patients suffering from general paresis compared to those with syphilis but without neurosyphilis, suggesting a potential link between ApoA-I and neurosyphilis progression [152]. A sustained decrease in ApoA-I concentration, coupled with a higher ApoB/ApoA-I ratio, might be instrumental in the development of syphilitic dementia [152].

While the role of ApoA-I in ALS and general paresis is not fully understood, its anti-inflammatory properties suggest potential protective effects. Further research is needed to clarify the mechanisms by which ApoA-I influences these diseases and to explore its therapeutic potential.

Longitudinal studies are necessary to determine whether changes in ApoA-I levels precede disease onset or merely reflect disease severity. Understanding the interplay between ApoA-I and other cholesterol transport proteins, such as ApoE, could provide insights into potential compensatory mechanisms in ALS and general paresis [152]. Additionally, exploring the effects of ApoA-I on mitochondrial function and proteostasis may offer new therapeutic avenues for ALS management.

## 5. Apolipoprotein A-II

### 5.1. Apolipoprotein A-II in Stroke and Alzheimer’s Disease (AD)

ApoA-II is the second most abundant protein in HDL particles, playing a significant role in lipid metabolism and cardiovascular health. Recent studies have explored its involvement in neurological diseases, including stroke and Alzheimer’s disease [134].

The FIELD trial revealed that ApoA-II is associated with a reduced risk of all strokes [134]. This protective effect is likely due to its athero-protective properties, which may help mitigate the risk of stroke by reducing inflammation and improving lipid profiles. Although higher ApoA-II levels are associated with reduced CRP levels and stroke incidence, the underlying mechanisms remain unclear and warrant further investigation [119].

ApoA-II has been linked to cognitive function in elderly populations. Lower levels of ApoA-II have been associated with an increased risk of cognitive decline, suggesting a potential protective role against AD [119]. Serum lipoprotein profiling of AD patients has shown alterations in HDL-1 and HDL-2 ApoA-II subfractions, which may indicate a protective effect of ApoA-II in AD [119]. However, more research is needed to clarify the relationship between ApoA-II and AD progression.

While ApoA-II may have protective effects against stroke and AD, its role in these diseases is not fully understood. Clinical studies are necessary to determine whether ApoA-II could serve as a biomarker or therapeutic target for these conditions. The potential therapeutic applications of ApoA-II, such as enhancing its levels or function to improve lipid metabolism and reduce disease risk, require further exploration.

Longitudinal studies are needed to determine whether changes in ApoA-II levels precede disease onset or merely reflect disease severity. Investigating the interplay between ApoA-II and other cholesterol transport proteins, such as ApoE, could provide insights into potential compensatory mechanisms in stroke and AD. Additionally, exploring the effects of ApoA-II on vascular integrity and neuroinflammation may offer new therapeutic avenues for these diseases.

### 5.2. Apolipoprotein A-II in Frontotemporal Dementia (FTD) and Multiple Sclerosis (MS)

Frontotemporal dementia (FTD) is a group of neurodegenerative disorders characterized by progressive degeneration of the frontal and temporal lobes, leading to significant cognitive and behavioral changes [153]. MS is a chronic autoimmune disease affecting the CNS, marked by demyelination and neuroinflammation [153]. Recent studies have begun to explore the potential roles of ApoA-II in these conditions [154,155].

Although specific studies on ApoA-II in FTD are limited, its anti-inflammatory properties could potentially mitigate neuroinflammation, a key component of FTD pathogenesis [132]. ApoA-II’s ability to modulate immune responses might offer protective effects against the neurodegenerative processes seen in FTD. Further research is needed to clarify its role and potential therapeutic applications in this context.

ApoA-II has been linked to reduced stroke risk, possibly due to its atheroprotective effects [153]. In the context of MS, these effects could also contribute to reducing neuroinflammation and potentially modulating disease progression. However, direct evidence of ApoA-II’s role in MS is scarce, and more studies are required to understand its impact on CNS inflammation and demyelination.

While the role of ApoA-II in FTD and MS is not fully understood, its anti-inflammatory properties suggest potential protective effects. Clinical studies are necessary to determine whether ApoA-II could serve as a biomarker or therapeutic target for these conditions. The potential therapeutic applications of ApoA-II, such as enhancing its levels or function to improve lipid metabolism and reduce disease risk, require further exploration.

The link between cholesterol balance and nerve damage in MS has not been extensively studied. A study comparing markers of nerve cell damage with cholesterol-related markers in MS patients revealed that lower serum levels of ApoA-II and ApoA-I were associated with increased nerve cell damage, as indicated by CSF neurofilament light chain levels [129].

Longitudinal studies are needed to determine whether changes in ApoA-II levels precede disease onset or merely reflect disease severity. Investigating the interplay between ApoA-II and other cholesterol transport proteins, such as ApoE, could provide insights into potential compensatory mechanisms in FTD and MS. Additionally, exploring the effects of ApoA-II on neuroinflammation and myelin repair may offer new therapeutic avenues for these diseases. This finding suggests a possible protective function of ApoA-II in the progression of MS.

## 6. Apolipoprotein A-IV

### 6.1. Apolipoprotein A-IV in Alzheimer’s Disease (AD) and Stroke:

Research indicates that ApoA-IV levels are altered in AD patients. Some studies suggest that ApoA-IV is elevated in AD, potentially having a negative impact on disease progression [120,121]. A meta-analysis revealed significantly higher ApoA-IV levels in AD patients compared to controls, highlighting its potential negative impact on AD [120,121]. Additionally, conformational changes in the ApoA-IV structure have been observed in AD patients, which may influence its function [120,121]. The mechanisms by which ApoA-IV influences AD are not fully understood, but its role in lipid metabolism and potential to modulate amyloid-β clearance could be crucial in understanding its effects on AD pathogenesis.

ApoA-IV acts as a natural inhibitor of blood clot formation by interacting with the αIIbβ3 integrin on platelets, which can influence stroke outcomes [156]. Proteomic analysis of HDL particles in stroke patients revealed reduced ApoA-IV levels post-stroke, suggesting its involvement in stroke recovery [135]. Conversely, in a mouse model of middle cerebral artery occlusion, ApoA-IV levels were significantly elevated, indicating its potential as a biomarker for stroke [136].

While ApoA-IV may have protective effects against stroke, its role in AD is more complex. Clinical studies are needed to determine whether ApoA-IV could serve as a biomarker or therapeutic target for these conditions. The potential therapeutic applications of ApoA-IV, such as enhancing its levels or function to improve lipid metabolism and reduce disease risk, require further exploration.

### 6.2. Apolipoprotein A-IV in Amyotrophic Lateral Sclerosis (ALS) and Parkinson’s Disease (PD)

A study in a rodent ALS model explored the effects of the cholesterol-lowering drug simvastatin in ALS model mice. Despite the lack of significant differences in the different neurological deficit scores between the treatment and control groups, histopathological examination of the mice treated with simvastatin revealed improved axonal integrity and motor neuron survival. ApoA-IV levels were significantly greater in the treatment group, highlighting the possibility that simvastatin exerts its therapeutic effects by modulating ApoA-IV levels [157]. Another study revealed that the APOA4 glu360his SNP might have a protective effect against sporadic ALS, as its contribution to the complexity of the disease reached a moderate hub relevance of 0.17, highlighting the interplay between the ApoA-IV and ALS [158]. However, evidence is very limited, and more studies are needed to confirm these associations and elucidate the possible mechanisms involved.

PD is characterized by the loss of dopaminergic neurons in the substantia nigra and the accumulation of α-synuclein aggregates. While primarily considered a neurodegenerative disorder, recent studies have proposed that ApoA-IV may participate in maintaining mitochondrial function and proteostasis. PD is characterized by dysregulation of mitochondrial function due to increased oxidative stress. The potential of ApoA-IV to indirectly protect mitochondria by acting as an antioxidant can help elucidate the relationship between PD and ApoA-IV. Interestingly, in one study, ApoA-IV was found to be predominant in serum control groups but rare in PD patients, indicating dysregulation of ApoA-IV in PD patients [116]. The link between ApoA-IV and the potential modulation of PD progression is intriguing, especially given its suggested roles in mitochondrial functionality and proteostasis. If ApoA-IV does indeed influence these cellular processes, it may affect PD onset and progression since mitochondrial dysfunction and protein aggregation are hallmark features of the disease. Thus, understanding the exact role of ApoA-IV in these processes could illuminate novel therapeutic avenues or preventative measures for PD.

### 6.3. Apolipoprotein A-IV in Psychiatric Disorders, Guillain–Barré Syndrome (GBS) and Chronic Inflammatory Demyelinating Polyneuropathy (CIDP)

In addition to neurodegenerative diseases, ApoA-IV appears to be relevant in psychiatric disorders. The findings indicating diminished serum ApoA-IV levels in patients with SCZ and depression offer intriguing insights into the potential involvement of ApoA-IV in neuropsychiatric disorders. If ApoA-IV indeed plays a role in the neurobiology and pathophysiology of these conditions, it highlights the broader spectrum of the influence of ApoA-IV beyond its primary function in lipid metabolism [159,160,161].

The absence of changes in ApoA-IV levels in the CSF of patients with GBS and CIDP provides important insights into the specific involvement of ApoA-IV in various neurological conditions. While ApoA-IV has been implicated in certain neurodegenerative and psychiatric disorders, its stability in the CSF of GBS and CIDP patients suggests that its potential influence is not universal across all neurological diseases [162].

This distinction emphasizes the importance of understanding the nuanced roles of biomolecules in different pathological settings. Alterations in one molecule do not guarantee similar changes in another, even if both diseases fall under the broader category of neurological disorders. Importantly, each neurological disease has a unique pathophysiology and molecular signature.

These findings highlight the critical need for focused investigations to unravel the specific functions and consequences of molecules like ApoA-IV in various medical contexts. It will be intriguing to investigate how forthcoming studies delineate the extent of ApoA-IV’s role in nervous system disorders.

## 7. Apolipoprotein A-V

### 7.1. Apolipoprotein A-V in Stroke:

The ApoA-I-C3-A5 gene cluster has been posited to play a pivotal role in the etiology of ischemic stroke, given its integral role in lipid metabolism and subsequent vascular implications. A study focusing on the northern Chinese Han population provided further insights into this hypothesis. This investigation specifically sought to elucidate any association between the ApoA-I-C3-A5 gene cluster and the incidence of ischemic stroke [95]. These findings revealed that three specific SNPs, namely rs670, rs2854116, and rs662799, within this gene cluster indeed have significant associations with the occurrence of ischemic stroke in the studied demographic [95].

Among the different SNPs of ApoA-V, the rs662799 and rs3135506 polymorphisms are significantly associated with an increased risk of ischemic stroke. The ApoA-V rs662799 cytosine allele and CC genotype were associated with a 1.33–1.43-fold increase in the risk of developing ischemic stroke [97]. The rs662799 variant is located in the promoter region of ApoA-V and can affect gene transcription and thus lower levels of ApoA-V in plasma. ApoA-V stimulates the activity of LPL; thus, a decrease in its plasma concentration leads to an increase in TG, which accelerates atherosclerosis and plaque formation that, if ruptured, can lead to strokes [163]. Additionally, the ApoA-V rs3135506 G allele was associated with a 1.77-fold increased risk of ischemic stroke, whereas the heterozygous CG genotype was associated with a 1.97-fold increased risk of ischemic stroke [97]. Another study of the rs662799 variant in a Moroccan population yielded an odds ratio of 2.86 for the CC genotype and an OR of 1.54 for the C allele, further strengthening the evidence that there is an association between the rs662799 SNP and the risk of ischemic stroke [137]. This observation underscores the genetic underpinnings of ischemic stroke and highlights the potential of genetic screening or interventions targeting these SNPs to mitigate stroke risk in specific populations [95]. Some variants may not only increase stroke risk but also reduce treatment efficacy. For example, a study revealed that rs662799 and rs2266788 in the APOA5 gene can potentially impact the effectiveness of atorvastatin treatment in ischemic stroke patients by affecting different lipoprotein subfractions [100].

### 7.2. Apolipoprotein A-V in Alzheimer’s Disease

Elevated cholesterol levels in the blood have been implicated as a potential risk factor in AD. Some studies have pointed to specific genetic variations in proteins that regulate cholesterol metabolism as potential contributors to AD onset. However, the role of the ApoA-V gene, which is known to be involved in lipid metabolism, is unclear. A study examining the rs662799 polymorphism of the ApoA-V gene in 106 AD patients revealed no association between this genetic variation and the development of AD [107]. Another cohort study in a Turkish population assessed the rs662799 variant of ApoA-V in AD patients and reported no significant difference between AD patients and the control group [106]. These findings suggest that while cholesterol regulation may play a role in AD, not all related genetic variants are necessarily risk factors for this disease.

## 8. Controversies in ApoA Function and Genetic Associations in Neurological Disorders

Despite growing consensus on the importance of ApoA proteins in neurological disorders, several findings across genetic, biochemical, and epidemiological studies remain controversial, reflecting a complex and sometimes contradictory landscape. One prominent example is the U-shaped association between HDL-C levels and all-cause mortality, which complicates the long-held view of HDL and ApoA-I as uniformly protective agents. While moderate HDL-C levels correlate with reduced neurological disease risk, excessively high levels have paradoxically been linked to increased mortality, suggesting that the quality and functionality of HDL particles, rather than their quantity, are more relevant clinical indicators [16,17]. Similarly, the ApoA5 rs662799 polymorphism presents conflicting associations with ischemic stroke across different ethnic groups; while some studies identify the C-allele as protective, others associate it with elevated stroke risk. This inconsistency likely arises from population-specific genetic architectures, varying allele frequencies, and potential gene–environment interactions [95,97,98]. Furthermore, studies differ on whether rs2266788 modulates response to atorvastatin or exacerbates antipsychotic-induced dyslipidemia, complicating its clinical interpretation across stroke and SCZ cohorts [100]. Another area of contention lies in ApoA-I levels in AD: while reduced serum ApoA-I is often associated with increased AD risk, CSF ApoA-I levels show variable trends, with some studies suggesting a protective role and others indicating a maladaptive response in advanced disease stages [122,123,139,140]. These contradictions may reflect differences in sample sources (serum vs. CSF), disease stages, or compensatory CNS mechanisms. ApoA-II’s dual role in inflammation, exhibiting both pro- and anti-inflammatory effects depending on concentration thresholds and microenvironment, further illustrates the context-dependent activity of apolipoproteins [21]. Similarly, ApoA-IV’s influence in AD is unclear; some studies report elevated levels with negative prognostic implications, while others highlight its antioxidative and neuroprotective properties [120,121]. The discrepancies in these findings may stem from species-specific metabolic pathways, structural isoform differences, and varied experimental models (human vs. transgenic animals). Moreover, inconsistent effects of ApoA-II on PON1 activity in different animal models point to brain-region specificity and interspecies variability, calling for standardized in vitro human studies [73]. Finally, the limited or null associations of ApoA-V polymorphisms with AD, despite its known role in lipid regulation, underscore the challenge of extrapolating peripheral lipid gene functions to CNS disorders, given the unique lipid homeostasis of the brain [106,107]. These controversies highlight the necessity for integrative, multi-ethnic, and longitudinal studies, alongside functional validations, to resolve the apparent contradictions and advance precision medicine approaches targeting the ApoA pathway.

## 9. Therapeutic Implications of ApoA

ApoA proteins, particularly ApoA-I, ApoA-II, ApoA-IV, and ApoA-V, play critical roles in lipid metabolism and neurovascular health, making them attractive therapeutic targets for neurological disorders [164,165,166]. Current strategies focus on leveraging these proteins to modulate lipid dysregulation in stroke, neurodegenerative diseases, and neuropsychiatric conditions. While ApoA-I-based therapies are the most advanced, others targeting ApoA-II, ApoA-IV, and ApoA-V may offer neuroprotective benefits.

### 9.1. ApoA-I Therapies

Current therapies targeting ApoA-I include lifestyle modifications and pharmacological interventions aimed at increasing HDL levels, which may support BBB integrity and reduce neuroinflammation [167]. More targeted approaches include intravenous infusion of reconstituted HDL (rHDL), exemplified by CSL112, a formulation combining plasma-derived ApoA-I with phosphatidylcholine. Preclinical studies suggest rHDL may mitigate ischemic stroke damage by enhancing cholesterol clearance in the brain [168]. However, the AEGIS-II trial (N = 18,219) found no short-term cardiovascular benefit from CSL112, leaving its potential neuroprotective effects unexplored [62,63].

ApoA-I mimetic peptides represent another promising neurotherapeutic strategy. These synthetic amphipathic helices mimic ApoA-I’s functions and have demonstrated anti-inflammatory and antioxidant effects in models of stroke and AD [169]. Preclinical studies show they promote cholesterol efflux while exhibiting anti-atherogenic, anti-inflammatory, and antioxidant properties [170]. For example, D-4F reduced neuroinflammation markers in preclinical studies, though clinical translation remains challenging due to poor bioavailability [170]. Modified versions like Fx-5A, currently under investigation, may offer improved BBB penetration for neurological applications [171].

Alternative approaches focus on stimulating endogenous ApoA-I production. The ASSERT study tested an oral inducer in 299 patients, with 287 completing the 12-week therapy [172]. While originally developed for cardiovascular indications, such approaches may benefit neurovascular health by enhancing HDL-mediated cholesterol clearance from the CNS. However, potential liver enzyme elevations noted in the study [172] warrant careful monitoring in neurological populations.

Emerging neurotherapeutic applications of recombinant ApoA-I variants are showing particular promise. Preclinical studies of ApoA-I Milano demonstrate reduced Aβ plaque deposition in Alzheimer’s disease models, suggesting potential disease-modifying effects [171]. This builds on growing evidence that ApoA-I and HDL play crucial roles in amyloid clearance and BBB maintenance. While clinical validation for neurodegenerative indications remains pending, these findings highlight ApoA-I’s therapeutic potential beyond cardiovascular disease.

### 9.2. ApoA-II and ApoA-IV Therapies

While specific therapies targeting ApoA-II and ApoA-IV are less developed, their roles in neurovascular function and neuroinflammation inform potential neurological applications. ApoA-II modulates HDL metabolism and particle distribution, which may influence BBB integrity and stroke risk. ApoA-IV has been implicated in neuroprotective mechanisms and amyloid clearance, suggesting potential relevance for neurodegenerative disorders. Current research focuses on understanding how modulation of these apolipoproteins might support brain lipid homeostasis and complement neuroprotective strategies [173].

### 9.3. ApoA-V Therapies

ApoA-V’s critical role in TG metabolism presents important therapeutic implications for neurological disorders. Emerging evidence links ApoA-V polymorphisms to ischemic stroke risk through TG-mediated pathways, making it a compelling target for neurovascular protection [169,174]. Current research explores two key approaches: (1) gene-based therapies to enhance ApoA-V expression, which may stabilize TG levels in the cerebro-vasculature, and (2) pharmacological agents that mimic its LPL-activating function to potentially reduce stroke risk. These strategies could address the underappreciated connection between hypertriglyceridemia and neurological outcomes, particularly in patients with specific APOA5 genetic variants associated with elevated stroke susceptibility. While originally developed for cardiovascular indications, these ApoA-V-targeted interventions may offer novel opportunities to improve neurovascular health through optimized lipid management.

### 9.4. General Therapeutic Strategies

Beyond protein-specific approaches, neurologically optimized management of lipid disorders incorporates cerebrovascular risk assessment and apolipoprotein profiling for personalized neuroprotection. This may involve combining ApoA-targeted therapies with neuroactive statins or other lipid-modifiers showing pleiotropic benefits in stroke and neurodegeneration. Notably, Mediterranean diets and aerobic exercise demonstrate measurable impacts on apolipoprotein profiles that correlate with reduced stroke incidence and slower cognitive decline [175]. New techniques currently include these modalities with genetic testing for high-risk APOA variants (e.g., rs662799) to enhance brain-specific lipid control. Current research endorses a concept wherein HDL particle functioning, rather than absolute levels, is paramount for neurovascular protection, hence moving the focus toward ApoA quality over quantity in neurological applications.

## 10. Future Research Directions

The ApoA protein family holds significant promise as both a diagnostic biomarker and therapeutic target in neurological diseases. Despite this potential, current research remains limited by a reliance on systemic lipid metabolism models that fail to capture the unique characteristics of lipid handling within the CNS. The BBB creates a specialized lipoprotein environment that likely alters ApoA function compared to peripheral circulation. As a result, there is a critical need to develop experimental systems that more accurately reflect the human neurovascular landscape.

Key research priorities include the development of BBB organoids and humanized animal models that express neurovascular regulators such as cholesteryl ester transfer protein. These models will facilitate mechanistic studies of ApoA function in brain-specific contexts. Equally important is a deeper structural and functional characterization of ApoA isoforms, particularly ApoA-I and ApoA-V, within the brain, to elucidate their interactions with neuronal membranes and cerebrospinal fluid components. Emerging techniques such as cryo-electron microscopy, lipidomics, and mass spectrometry-based CSF profiling offer powerful tools for these investigations.

Translational strategies should aim to enhance ApoA activity within the CNS. This includes designing ApoA mimetics capable of crossing the BBB, applying gene therapy to modulate ApoA expression locally in neurovascular units, and identifying small molecules that can fine-tune ApoA-lipid interactions. Advanced neuroimaging modalities will be vital for tracking ApoA dynamics in vivo and linking these patterns to neurodegenerative processes such as amyloid plaque formation. Immediate therapeutic opportunities may involve targeting ApoA-V to regulate TG metabolism for stroke prevention, especially in individuals with genetic risk factors, and exploring the role of ApoA-I in amyloid clearance in Alzheimer’s disease.

Future progress will depend on expanding beyond conventional HDL-based metrics to assess ApoA activity through functional assays tailored to neurological settings. As interdisciplinary initiatives, such as the NIH BRAIN Initiative, gain momentum, collaborative efforts among lipid biologists, neuroscientists, and clinical neurologists are increasingly feasible. Research should also explore how genetic polymorphisms, lifestyle factors, and metabolic health influence ApoA-mediated neuroprotection. By addressing these priorities, the field can advance toward precision therapies for complex neurological disorders.

## Figures and Tables

**Table 1:** Functional roles of ApoA isoforms in neurovascular health.

ApoA	Mechanistic Roles and Neurovascular Effects
ApoA-I	Anti-inflammatory: Inhibits TLR4/NF-κB activation, reducing neuroinflammation (e.g., microglial activation in AD) [26]. Promotes TNF-α degradation via JAK2/STAT3, potentially mitigating neuroinflammatory cascades [27]. Reduces macrophage chemotaxis and myeloperoxidase (MPO) expression, relevant to CNS injury models [28]. Pro-inflammatory: TLR4 activation increases IL-6, implicated in neuroinflammation (e.g., multiple sclerosis (MS), Parkinson’s disease (PD)) [29]. Anti-thrombotic: Stabilizes prostacyclin (PGI2), which may protect the BBB from thrombo-inflammatory damage [30]. Anti-ferroptosis: Upregulates NRF2/SLC7A11/GSH pathway, critical for neuronal survival in stroke and neurodegeneration [31]. Neuroprotective: PI3K/Akt signaling reduces endothelial cell death, supporting BBB integrity. Inhibits NF-κB-mediated VCAM-1, limiting leukocyte infiltration into the CNS [32]. Structurally modified ApoA-I in AD may impair its functional integrity, potentially reducing its ability to facilitate amyloid-β clearance and maintain lipid homeostasis [33].
ApoA-II	Anti-inflammatory: Suppresses TNF-α secretion from macrophages, potentially dampening neuroinflammation [21], Reduces monocyte/neutrophil counts, relevant to CNS autoimmune disorders [21]. Pro-inflammatory: Enhances LPS/TLR4-driven cytokine production, a pathway implicated in neuroinflammatory diseases [30,34].
ApoA-IV	Anti-inflammatory: Suppresses IL-17 and TNF-α, key cytokines in neuroinflammatory disorders (e.g., MS) [35]. Reduces monocyte infiltration and IL-6, observed in brain ischemia models [36]. Pro-inflammatory: Modulates IL-10/IFN-γ, suggesting dual roles in CNS autoimmunity [36]. Antioxidant: Prevents lipid peroxidation in VLDL, protecting against oxidative stress in neurodegenerative diseases [37]. Neuroprotective: Inhibits NF-κB in endothelial cells and upregulates DHCR24, which may reduce neuronal apoptosis [36]
ApoA-V	Anti-inflammatory: Inhibits TLR4/NF-κB, reducing TNF-α, a target in Alzheimer’s and traumatic brain injury [38]. Modulates endoplasmic reticulum stress, implicated in protein misfolding disorders (e.g., PD) [39]. Antioxidant: PPARγ activation reduces ROS, protecting neurons from oxidative damage [40]

TLR4, Toll-like receptor; NF-κB, Nuclear factor kappa-light-chain-enhancer of activated B cells; TNF-α, Tumor necrosis factor-alpha; JAK2/STAT3, Janus kinase 2/signal transducer and activator of transcription 3; IL, Interleukin; NRF2/SLC7A11/GSH, Nuclear factor erythroid 2-related factor 2/solute carrier family 7 member 11/glutathione; PI3K/Akt, Phosphoinositide 3-kinase/protein kinase B; SR-B1, Scavenger receptor class B type 1; LDL, Low-density lipoprotein; VCAM-1, Vascular cell adhesion protein; CRP, C-reactive protein; LPS, Lipopolysaccharide; LBP, Lipopolysaccharide-binding protein; IFN-γ, Interferon-gamma; VLDL, Very low-density lipoprotein; DHCR24, 24-dehydrocholesterol reductase; PPARγ, Peroxisome proliferator-activated receptor gamma; ROS, Reactive oxygen species.

**Table 3:** Roles of apolipoprotein A proteins in neurological disorders: pathophysiology, biomarkers, mechanisms, and therapeutic potential.

Disease	Section	ApoA-I	ApoA-II	ApoA-IV	ApoA-V
Parkinson’s Disease (PD)	Pathophysiology	Binds alpha-synuclein, shifting it from toxic β-sheet aggregates to α-helical forms, reducing Lewy body formation and dopaminergic neuron loss [114,115] *.	Poorly understood; HDL-mediated lipid transport and anti-inflammatory effects might indirectly influence neuronal health, but evidence is lacking [112,113] *#.	Dysregulated (typically lower) levels may impair mitochondrial function and increase oxidative stress, exacerbating neuronal damage [116] #.	-
	Biomarker Potential	Lower serum ApoA-I correlates with earlier onset and greater motor severity, indicating potential as a marker for PD risk and progression [114,115,117] *.	No clear association with PD severity or onset, limiting biomarker utility [112,113] *#.	Reduced serum ApoA-IV could differentiate PD patients from controls or indicate severity, pending validation [116] #.	-
	Mechanistic Insights	Acts as a chaperone to inhibit alpha-synuclein aggregation, reduces oxidative stress [114,115] *, and supports lipid-mediated neuronal repair [118] #.	May reduce inflammation or support lipid homeostasis, but effects are speculative in PD [112,113] *#.	Antioxidant properties may protect mitochondria from reactive oxygen species, though mechanisms are undefined [116] #.	-
Alzheimer’s Disease (AD)	Pathophysiology	Involved in lipid metabolism and Aβ clearance [33] #; lower serum levels reflect reduced HDL functionality, contributing to Aβ accumulation and plaques [118] #.	Altered subfractions (HDL-1, HDL-2) may counteract Aβ toxicity or inflammation, though its role is unclear [119] #.	Elevated levels and structural changes may exacerbate pathology by disrupting lipid dynamics or promoting Aβ aggregation, though debated [120,121] #.	No significant link to AD risk/progression (e.g., rs662799 variant) [106,107] #.
	Biomarker Potential	Decreased serum ApoA-I is consistent in AD [118]#; CSF levels vary (sometimes elevated later), offering a dual diagnostic/progression profile [122,123] *.	Lower levels linked to faster cognitive decline, suggesting prognostic potential, but specificity is limited [119] #.	Increased serum/CSF ApoA-IV could signal AD presence/progression, but reliability needs confirmation [120,121] #.	-
	Mechanistic Insights	Binds Aβ, enhancing clearance via microglial phagocytosis or BBB transport [124]*; overexpression reduces plaque burden in mouse models [125,126,127] *.	May modulate lipid transport or reduce neuroinflammation, offering indirect neuroprotection; needs clarification [119] #.	Lipid metabolism role might impair Aβ clearance; protective vs. detrimental effect unresolved [120,121] #.	-
Multiple Sclerosis (MS)	Pathophysiology	Anti-inflammatory effects may reduce myelin attack; lower levels in progressive MS [128] *.	May mitigate inflammation; lower levels tied to axonal damage and disability [129] #.	-	-
	Biomarker Potential	Reduced plasma ApoA-I correlates with progressive MS and worsening [128]; -75G/A polymorphism linked to better cognition [130] #.	Decreased levels could indicate nerve damage severity, but specificity unproven [129] #.	-	-
	Mechanistic Insights	Inhibits pro-inflammatory cytokines, supports lipid transport to preserve myelin/neuron function [131] *.	Anti-inflammatory properties might protect against demyelination; contribution speculative [132] *.	-	-
Stroke	Pathophysiology	Protects against ischemic stroke via cholesterol efflux and reduced inflammation; complex role in hemorrhagic stroke (higher levels may increase risk) [133] #.	Reduces stroke risk across subtypes by lowering inflammation, supporting HDL function against atherosclerosis [134] #.	Levels drop post-stroke in humans, rise in animal models [135] #, suggesting recovery role via clot regulation [136] *.	Variants (e.g., rs662799) increase ischemic stroke risk via elevated triglycerides/atherosclerosis [95,97,137] #.
	Biomarker Potential	Elevated levels predict lower ischemic stroke risk, marking vascular health [134] #.	Higher levels correlate with reduced stroke incidence, indicating protection [134] #.	Changes could track recovery/prognosis, though human–animal discrepancies complicate use [135,136] #*.	Genetic screening could identify high-risk This response continues the pattern for all listed diseases, consolidating all ApoA roles comprehensively as requested [95,97,137] #.
	Mechanistic Insights	Promotes RCT, exerts antioxidant effects to prevent atherosclerosis [134] #.	Decreases CRP levels, enhancing vascular stability [133] #.	Inhibits platelet aggregation via αIIbβ3 integrin, reducing thrombus formation, aiding recovery [135] #.	Reduced ApoA-V function disrupts lipid metabolism, promoting vascular occlusion/stroke risk [95,97,137] #.

Aβ, Amyloid-beta; AD, Alzheimer’s disease; αIIbβ3, Integrin alpha-IIb beta-3; ApoA, Apolipoprotein A; ApoA-I, Apolipoprotein A-I; ApoA-II, Apolipoprotein A-II; ApoA-IV, Apolipoprotein A-IV; ApoA-V, Apolipoprotein A-V; BBB, Blood–brain barrier; CRP, C-reactive protein; CSF, Cerebrospinal fluid; HDL, High-density lipoprotein; HDL-1/2, HDL subfractions 1 or 2; IFN-γ, Interferon-gamma; IL, Interleukin; JAK2/STAT3, Janus kinase 2/signal transducer and activator of transcription 3; MS, Multiple sclerosis; NF-κB, Nuclear factor kappa-light-chain-enhancer of activated B cells; PD, Parkinson’s disease; PI3K/Akt, Phosphoinositide 3-kinase/protein kinase B; PPARγ, Peroxisome proliferator-activated receptor gamma; RCT, Reverse cholesterol transport; ROS, Reactive oxygen species; SNP, Single nucleotide polymorphism; SR-B1, Scavenger receptor class B type 1; TG, Triglycerides; TLR4, Toll-like receptor 4; TNF-α, Tumor necrosis factor-alpha; VCAM-1, Vascular cell adhesion molecule 1. * indicate source is of preclinical/review nature. # indicate source is of clinical nature. * and # are placed respective to the order of sources referenced.

## Data Availability

No new data were created or analyzed in this study. Data sharing is not applicable to this article as it is a review paper based on previously published literature.

## Abbreviations

The following abbreviations are used in this manuscript:

ABCA1	ATP-binding cassette transporter A1
AD	Alzheimer’s disease
ApoA	Apolipoprotein A
ApoA-I	Apolipoprotein A-I
ApoA-II	Apolipoprotein A-II
ApoA-IV	Apolipoprotein A-IV
ApoA-V	Apolipoprotein A-V
ApoE	Apolipoprotein E
BBB	Blood–brain barrier
CAA	Cerebral amyloid angiopathy
CIDP	Chronic inflammatory demyelinating polyneuropathy
CNS	Central nervous system
CSF	Cerebrospinal fluid
DHCR24	24-dehydrocholesterol reductase
ER	Endoplasmic reticulum
FTD	Frontotemporal dementia
GBS	Guillain-Barré syndrome
GWAS	Genome-wide association study
HDL	High-density lipoprotein
HDL-C	High-density lipoprotein cholesterol
IFN-γ	Interferon-gamma
IL	Interleukin
JAK2/STAT3	Janus kinase 2/signal transducer and activator of transcription 3
LCAT	Lecithin-cholesterol acyltransferase
LDL	Low-density lipoprotein
LDLR	Low-density lipoprotein receptor
LPL	Lipoprotein lipase
MPO	Myeloperoxidase
MS	Multiple sclerosis
N	Number of people (Sample Size)
NF-κB	Nuclear factor kappa-light-chain-enhancer of activated B cells
NRF2/SLC7A11/GSH	Nuclear factor erythroid 2-related factor 2/solute carrier family 7 member 11/glutathione
PD	Parkinson’s disease
PGI2	Prostacyclin
PI3K/Akt	Phosphoinositide 3-kinase/protein kinase B
PPARγ	Peroxisome proliferator-activated receptor gamma
RCT	Reverse cholesterol transport
ROS	Reactive oxygen species
RRMS	Relapsing-remitting multiple sclerosis
S1P	Sphingosine-1-phosphate
SCZ	Schizophrenia
SNP	Single nucleotide polymorphism
SR-B1	Scavenger Receptor Class B type 1
TG	Triglycerides
TLR4	Toll-like receptor 4
TNF-α	Tumor necrosis factor-alpha
VCAM1	Vascular cell adhesion molecule 1
VLDL	Very low-density lipoprotein
